# Newly qualified midwives’ perceptions of their level of midwifery clinical competence during community service in KwaZulu-Natal, South Africa

**DOI:** 10.4102/hsag.v26i0.1670

**Published:** 2021-10-28

**Authors:** Amanda Ngcobo, Olivia B. Baloyi, Mary Ann Jarvis

**Affiliations:** 1 Discipline of Nursing, College of Health Sciences, University of KwaZulu-Natal, Durban, South Africa

**Keywords:** community service, competence, midwifery, new qualified midwives, perceptions

## Abstract

**Background:**

There are links between the inadequate numbers of competent midwives and high maternal mortality ratios and neonatal mortality rates which highlights the significance of job-ready, newly qualified midwives who can display clinical competence. The South African Nursing Council regulated mandatory community service, as a supportive year to develop clinical competence.

**Aim:**

To explore and describe newly qualified midwives’ perceived level of midwifery clinical competence during community service.

**Setting:**

Both the event of the pandemic and the distribution of the potential participants across various geographical settings necessitated planning for data collection in real and online settings convenient to them.

**Method:**

Non-probability purposive sampling was utilized to select and invite the post community service, newly qualified midwives (*N* = 65), of the select university, who underwent community service in 2018 and experienced exposure to maternity care settings in the eThekwini District (*n* = 23). Data were collected through five focus groups and analysed through Elo and Kyngas’ content analysis.

**Results:**

Three categories emerged: transitioning from the sheltered education environment to the real practice world, support in practice: disparate realities and interception of mentorship. Six subcategories accompanied the categories.

**Conclusion:**

Newly qualified midwives’ clinical confidence and competence transitioning from the safe academic environment to the authentic accountable clinical setting hinges on mentorship and welcoming, non-stigmatising supportive relationships that facilitate the integration of previous learnings into community service practice.

**Contribution:**

The study allows for audibility and awareness of the transitioning midwives’ perceptions highlighting the significance to maternity staff and policy makers, of supportive relationships and structures.

## Introduction

Global efforts to decrease maternal mortality ratio (MMR) and neonatal mortality rate (NMR) reflect every woman’s right to the best possible healthcare during pregnancy and childbirth (World Health Organisation [WHO] 2011). In sub-Saharan Africa, the MMR remains inequitably high, although there is some evidence of changes (WHO 2019). For example, since 2016, South Africa has witnessed a decline in MMR by 2.46 per 100 000 live births to 119.0 per 100 000 live births (WHO 2019); yet within this landscape of progress, one of its provinces, KwaZulu-Natal, had MMR of 124.9 per 100 000 births (KwaZulu-Natal Department of Health [KZNDoH] 2018a). High MMR have links to inadequate numbers of competent midwives (WHO 2019) highlighting the significance of job-ready, newly qualified midwives who can display clinical competence (Nentshisaulu & Maputle 2018).

Competence is defined by the South African Nursing Council (SANC) as: ‘the combination of knowledge, psychomotor, communication and decision-making skills that enable an individual to perform’ (SANC 2021). Further expansion to the definition is achieved by adding the phrase ‘within the defined scope of practice at an acceptable level of proficiency’ (Singapore Nursing Board 2018:1). The definition of competence is not static and defined differently over a professional’s lifetime (Benner 1984). The midwife moves back and forth, from being novice to expert (Benner 1984), as technology advances and knowledge evolves. When individuals have initially acquired a skill (whether cognitive or practical), it needs reinforcing to maintain a similar competence level. A skilled midwife, according to the International Confederation of Midwives (2010) is:

[*E*]ducated and trained to proficiency in the skills needed to manage normal (uncomplicated) pregnancies, childbirth and the immediate postnatal period, and in the identification, management and referral of complications in women and new-borns. (p. 6)

In lower-middle-income countries (LMICs), including South Africa, the midwife’s workload demands are increased by the fluid healthcare system (Baloyi & Mtshali 2018). Furthermore, the healthcare systems in LMIC are characterised by complicated and complex maternity cases, the high risk profile of pregnant women, and MMR’s high burden (Baloyi & Mtshali 2018; Van Graan & Williams 2017; WHO 2019). The complexity of the maternity cases in LMICs requires higher-order thinking skills, facilitating an ability to think critically and problem-solving by applying clinical reasoning skills, thereby enabling informed clinical decisions (Baloyi & Mtshali 2018; Levett-Jones et al. 2012; Van Graan & Williams 2017). Newly qualified midwives who are job-ready need to demonstrate capability in providing evidence-based care, practice independently and be accountable for their actions (Netshisaulu & Maputle 2018).

In South Africa, the nursing regulatory body, the SANC, recognised the need for a pre-licensure mentorship period before registration as a general nurse and midwife (RSA 2006). Mandatory community service was legislated, with one of its goals to expand the newly qualified practitioners’ level of clinical competence (Govender, Bhengu & Brysiewicz 2015; RSA 2006). Although newly qualified midwives are accountable practitioners at this point, they may not be fully conversant with the wide range of skills required to do their job effectively (Black 2018). Post qualification as a nurse and midwife, new graduates spend a mandatory full year in community service in Department of Healthcare settings, working under the supervision of a licenced nurse midwife. In the pre-licensure period, licenced midwives can support the new graduate in competence development (Benner 1984) and higher-order thinking skills (Baloyi & Mtshali 2018).

The transition period from either a university or college setting to the world of work is stressful and challenging for the new graduate because of the failure in meeting the new role’s expectations (AI Awaisi, Cooke & Pryjmachuk 2015). Often the newly qualified midwife is left feeling incompetent (AI Awaisi et al. 2015; Freeling & Parker 2015; Shongwe 2018), and in the nursing profession, this lack of practice readiness has become an area of concern (Strauss et al. 2016). Nursing education programmes, formal support structures and the working environment seem to influence the transition experienced by newly qualified nurses and midwives to practice (AI Awaisi et al. 2015). It remains unknown whether the knowledge or skills acquired during midwifery education are adequate. Understanding the newly qualified midwives’ views regarding their midwifery clinical competence may help prepare midwives for their transition.

This study sought to explore newly qualified midwives’ perceptions of their level of midwifery clinical competence during community service in eThekwini District, KwaZulu-Natal. The researchers envisaged that findings could inform the review of structures and standards of community service to improve the newly qualified midwives’ job performance and help to inform the education provided in their pre-service undergraduate midwifery education.

### Aim

The study aimed to explore and describe newly qualified midwives’ perceived level of midwifery clinical competence during community service.

## Research design and method

### Study design and approach

A social constructivism paradigm guided the explorative and descriptive qualitative approach of this study. The approach was appropriate as it granted the researchers an opportunity to engage in discussions with the participants and gain a deeper understanding (Crotty 1998) about their level of midwifery clinical competence during community service.

### Study setting

Both the event of the pandemic and the distribution of the potential participants across various geographical settings necessitated planning for data collection in real and online settings convenient to them. The study took place with the newly qualified graduates of a university located in KwaZulu-Natal, South Africa, which offers a problem-based, competency-oriented and student-centred Bachelor of Nursing Degree (Mthembu, Mtshali & Frantz 2014). In South Africa, nursing and midwifery are part of the same programme, where nursing content influences midwifery knowledge and skills, qualifying the graduate as a nurse and midwife. The programme’s goal is to contribute life-long learners to the pool of nurses and midwives regulated by the (SANC 2016b). The university engages the students in innovative teaching and learning strategies such as flipped classrooms, online learning, reflective journaling and simulated learning to facilitate higher-order thinking skills, as they acquire SANC competencies ready for clinical practise, commencing in community service. During community service, students are placed at various Department of Health facilities, ranging from clinics to community health centres to hospitals and within these facilities they are rotated through the units.

Amongst other geographical areas, the new graduates carry out the mandatory community service in the eThekwini District of KwaZulu-Natal province, South Africa. The district continues to present with a high MMR of 101 per 100 000 live births (KZNDoH, 2018b), compared with 70 per 100 000 live births targeted in the Sustainable Development Goal (SDG)-3.1 (UNSD 2018). The NMR in the eThekwini District is 21 per 1000 live births (KZNDoH, 2018b), compared with the targeted SDG-3.2 of 12 per 1000 live births (UNSD 2018).

### Study participants and sampling

Non-probability purposive sampling (Etikan & Bala 2017) was utilised to select and invite the newly qualified midwives’ (*N* = 65) of the select university, targeting those who underwent community service in 2018 and experienced maternity exposure in the eThekwini District (*n* = 23). The community service graduates of the select university belonged to the same WhatsApp group. The researcher (hereinafter refers to A.N.), a recent Bachelor of Nursing graduate from the select university, had permission from the WhatsApp group administrator to post an invitation, with study inclusion criteria, to the potential participants. The researcher provided her contact details so that if they met the inclusion criterion of working in maternity units in the eThekwini District during community service in 2018, they could respond to her directly. One potential participant declined, without providing a reason, through a WhatsApp message to the researcher, resulting in a sample size of 22 participants. This method allowed for the invitation of participants who were well informed about the phenomenon under discussion. In addition, the invited number of potential participants implied the anticipated data saturation.

### Data collection

Prior to data collection, researcher conducted a pilot focus group with fellow master students with no amendments to the questions but feedback to modify the questioning style to allow for probing and a need for the researcher to bracket (Tuckett 2005). Data collection commenced on 27 November 2019, for 6 months. The participants’ availability and location (face-to-face focus groups) determined where data were collected and their division into five focus groups. The face-to-face focus groups (*n* = 3), were led by the researcher and another Masters student (S.X.). However, the COVID-19 pandemic regulations (Hedding et al. 2020) necessitated a change from face-to-face focus groups to online focus groups (*n* = 2). The online focus groups were conducted by A.N., and research supervisors, M.A.J. and O.B.B., PhD graduates experienced in qualitative research. All participants provided written consent and agreed to the audio recording. The five focus groups were closed groups, which consisted of between four and five participants and the recording lasted between 22 min and 36 min. The fourth focus group evidenced data saturation and the last focus group served to ensure that no new categories emerged from the data. An interview guide was used with one open-ended question, followed by five probing questions (Tausch & Menold 2016). The interview commenced with an open-ended question that read as follows: ‘What were your perceptions regarding your midwifery clinical competence as NQMs doing community service in eThekwini District KwaZulu-Natal?’

### Data analysis

All three researchers (A.N., O.B.B. and M.A.J.) manually coded and analysed the data, which had been transcribed verbatim from the audio recordings of the five focus groups. Content analysis performed by Elo and Kyngas (2008) was used. This systematic technique compresses many text words into fewer content categories, following the stages of preparation, organising and reporting of the data (Elo & Kyngas 2008). Simultaneous to data collection to allow for evidence of saturation, data from the five focus groups were analysed through several meetings between the researchers.

### Measures of trustworthiness

The researchers ensured trustworthiness by following the criteria of credibility, transferability, dependability and confirmability as outlined by Shenton (2004). Data verification occurred through both face-to-face contacts and the participants’ permission by sending the transcripts via e-mails, with no need for corrections, followed by groups to allow for member checking (Shenton 2004). In addition, the researcher piloting the interview guide with constructive feedback, contributed to credibility. The description of the study setting involving post community service midwives in a South African context and the described qualitative methodology allows for transferability. The generation of an audit trail and the use of participant quotes to support interpretation, ensured confirmability and the certainty of the accuracy, relevance and meaning of data (Shenton 2004). The audit trail included audio recording and verbatim transcription of the focus group discussions, written descriptive field notes, the fourth focus group achieving data saturation and independent data coding followed by discussions to reach solidarity on the gathered findings (Shenton 2004).

### Ethical considerations

The study design adhered to all applicable ethical principles. The Research Ethics Committee of the study university provided clearance (reference number: HSSREC/00000306/2019). Face-to-face data were gathered from the focus groups, in real or online locations convenient to the participants, aligning with the ethical principles of respect and non-beneficence (Lulé, Kübler & Ludolph 2019). Further to the principle of non-beneficence, participants in the online groups were reimbursed for their data costs (Lulé et al. 2019). In both the face-to-face and online focus groups, all participants verbally agreed to confidentiality of information shared. Participants in the online focus groups were required to switch off their web-cameras to maintain anonymity and select pseudonyms as online identifiers (Sugiura, Wiles & Pope 2017). According to Section 18 of the POPI-A Act, the researcher should discuss with the data subjects the purpose of collecting personal information (Staunton et al. 2020). Therefore, through the WhatsApp group administrator, the researcher requested the potential participants’ e-mail addresses to send them the information sheet, which explained the purpose of the study and the researcher’s role, consent form and provided 3 days to consider participation.

## Results

### Profile of the participants

Twenty-two (22) newly qualified midwives (*n* = 6 males; *n* = 16 females) ranging between 24 to 32 years of age (mean = 25.4 years) participated in the study (Table 1). The participants had 1-year of community service experience (2018) and worked in maternity units in the eThekwini District.

**TABLE 1:** Demographic profile of participants (*n* = 22).

Format	Focus group (FG) and date conducted	Participant number	Age (years)	Gender
Face-to-face	FG1	P1	24	Female
	27 November 2019	P2	24	Male
		P3	25	Male
		P4	30	Female
	FG2	P1	25	Male
	14 December 2019	P2	25	Male
		P3	25	Female
		P4	25	Female
		P5	25	Female
	FG3	P1	25	Female
	18 December 2019	P2	25	Female
		P3	24	Female
		P4	25	Male
		P5	32	Female
On-line	FG4	P1	25	Female
	26 May 2020	P2	25	Female
		P3	25	Female
		P4	25	Male
	FG5	P1	25	Female
	06 June 2020	P2	25	Female
		P3	25	Female
		P4	25	Female

### Study’s findings

Three categories and six subcategories emerged from the data following content analysis (Table 2). Participant quotes have been left verbatim with grammatical errors to preserve the participants’ voices.

**TABLE 2:** Categories and subcategories following content analysis.

Categories	Subcategories
1. Transitioning from the sheltered education environment to the real practice world	1.1. Transitional insecurities 1.2. Unrelatable clinical education and assessments
2. Support in practice: Disparate realities	2.1. The luck of the draw 2.2. External stigmatisation limits support
3. Interception of mentorship	3.1. Under-resourced 3.2. Active contemplation of community service goals

### Category 1: Transitioning from the sheltered education environment to the real practice world

The transition from the university’s known familiar environment, which encompassed a student’s role to the less familiar world of work in a novice practitioner’s role, resulted in the newly qualified midwives expressing their concerns regarding moving into their new roles and unfamiliar settings.

#### Subcategory 1.1: Transitional insecurities

Participants highlighted that they had trouble in dealing with the feelings of fear, nervousness and associated anxiety at the beginning of their community service. The feelings of insecurity occurred as they lost their student status with its accompanying roles enshrined in the sheltered university environment and transitioned to the real world of professional practice in the maternity units. Participants highlighted the following:

‘…Transitioning into the real world of practice brought in me so much insecurities.’ (FG4, P1, Female)

‘…Like we were so used to the university environment, which offered safety for us now I was so anxious ….’ (FG2, P2, Male)

‘… We have always been under an umbrella of the university so … we felt like we were always protected.’ (FG4, P4, Male)

‘… being in the real practice world of a midwife brought in me fear, anxiety like I was always nervous.’ (FG4, P3, Female)

Furthermore, the inherent anxieties and insecurities as experienced by the participants in the role adaptation were triggered by the need for independent practice, unlike their previous experience of being supervised as students, which is evident in the following extracts:

‘As a student one always worked under the supervision of a registered midwife … now things had changed, I was on my own I had to prove myself in my new role as an independent practitioner … but I was scared I had so much insecurities about myself and I had a lot of anxiety.’ (FG2, P2, Male)

‘Adapting into the new role of being an independent midwife was not an easy one … studenthood does not expose us to that independence that is expected of us when we graduate, we were always supervised … the reality is now one needs to prove that I can do it on my own and because I was not used to it for four years now I was really … really insecure in most of the things I did as a midwife.’ (FG4, P3, Female)

#### Subcategory 1.2: Unrelatable clinical education and assessments

Participants acknowledged being well equipped theoretically. However, they expressed feeling incompetent in performing specific midwifery procedures in the clinical setting, as illustrated in the following extracts:

‘… I had the theory but in terms of confidence and … and the ability to go in there, actually perform the deliveries and all that it was a bit challenging, I felt so incompetent clinically.’ (FG1, P3, Male)

‘The university prepared us well theoretically…but when it comes to clinical skills I don’t think this area was given much attention like the theory was…yet the feeling of incompetency in me …’ (FG3, P1, Female)

‘… I think it is well known that theoretically university students are good … as a university graduate yes this is true…but the clinical component was not as good, and this left us as university students feeling so incompetent in the clinical field.’ (FG4, P1, Female)

This level of incompetence in clinical practice was attributed to the disparity between the simulated learning strategies that were used for clinical skills practice and assessment during university education and the real-life setting. In a simulated setting, participants explained the use of mannequins for their objective structured clinical examinations (OSCEs), which they found to be quite different and without the complexities they were exposed to in the real-life setting. As a result, participants expressed a lack of confidence in their community service as indicated here:

‘…The clinical skills laboratory provided that safe environment for us to practice, which was okay but for me as a person I think the problem was that the simulation did not resemble what happens in the actual clinical practice.’ (FG4, P3, Female)

‘As a result it was difficult to unlearn or switch off from what was taught in the clinical skills laboratory.’ (FG3, P1, Female)

‘We did all our clinical examinations like OSCE in the clinical skills laboratory…and mostly we used mannequins…whatever procedure we had to do like managing PPH or shoulder dystocia was in a form of acting and in the real-world practice this is not the same the patient is there and this is an emergency one needs to think fast and know what they are doing.’ (FG5, P5, Female)

‘…Hence I feel the clinical skills laboratory did not fully prepare us for the real-world practice.’ (FG3, P3, Female)

### Category 2: Support in practice: Disparate realities

The second category showed that support in practice depended on who the participants were placed with during community service. This second category had two subcategories.

#### Subcategory 2.1: The luck of the draw

The idiom ‘the luck of the draw’ describes the participants’ perception of their allocation as one of chance. The participants shared differences about the support they received, which appeared to depend on their setting and the staff’s willingness. They revealed that they were either supported with a ‘hand-holding, nurturing’ approach or an ‘adapt or die’ approach. Here is how some of the participants verbalised this finding:

‘…The support we got from our different clinical facilities was not consistent … it was more on a luck basis if I may out it that way sometimes one would get staff that welcoming with warm hands, the staff that will be willing to help you whenever you need help, shuuuuu but there will be those Sisters I don’t know if it jealousy or what, but whenever you ask something from some of the Sisters they’ll just say that … “Oh you don’t know this”… they’ll tell you that you still fresh from college, so you supposed to know these things ….’ (FG2, P4, Female)

‘… My first exposure during community service was in the labour ward there was this one midwife she was so good, she supported me so well she was my shoulder to cry on … I kind of liked midwifery because of her … Oh God then I had to rotate to postnatal … the staff there was so bad they treated me so bad they did not want to support me in anyway but just constantly reminded me of my degree and how I must show it off then I started disliking midwifery.’ (FG1, P4, Female)

However, the participants acknowledged that the initial ‘hand-holding’ of a willing mentor was helpful and provided a safety net to build their confidence levels. This is how the participants expressed their views concerning the initial hand-holding:

‘… In addition to what my colleagues said I think for me the initial support like hand-holding someone being there for you all the times was the best that’s how I developed my confidence in midwifery.’ (FG1, P2, Male)

‘… One always felt safe and confident when working with someone who was willing to offer help and support unconditionally … like some people would offer themselves to support you I really appreciated that.’ (FG2, P1, Male)

#### Subcategory 2.2: External stigmatisation limits support

The newly qualified midwives expressed that they were perceived as ‘all-knowing’ with some of the senior staff expecting that they perform and know all midwifery procedures with little consideration of their previous exposure to midwifery in their university education.

‘… The stigma attached to us as university students made it very difficult for us … we not supported to the fullest. We were always reminded of being degree products and that we should know everything it doesn’t matter if you have seen before or not as a degree student you must know it.’ (FG4, P3, Female)

‘… Being a degree product is like a crime even the senior midwives the people we look upon for support they stigmatise us…instead of them supporting us they constantly remind us of our degree, the theory we have and all, yet they failed to fully support us.’ (FG3, P3, Female)

As ‘all-knowing’ was the expectation on the part of senior colleagues, the participants were reluctant to request assistance as they perceived they would demonstrate their lack of knowledge or lack of practical ability. The fear displayed by the participants was predominantly because senior colleagues expected the newly qualified midwives to have learnt or been exposed to all midwifery procedures by holding a Bachelor’s degree. A method of coping was to pretend they knew the information.

‘… It was very difficult to ask for help…I expected senior midwives to avail themselves to support and show me around … I just kept everything to myself because I felt asking for help would sell me out as someone who doesn’t know what she is doing.’ (FG1, P4, Female)

‘… Because they expected me to know everything I pretended as though I know everything and did not ask for any help if no one came forward to help me … I was tired of exposing and humiliating myself.’ (FG2, P4, Female)

‘I used to get scared to ask questions sometimes because the staff there … they would say “no you … with a bachelor’s degree so you know everything eeh … they will just assume that you know everything. So, when … whenever you like ask … they’ll just give you that look….”’ (FG5, P2, Female)

### Category 3: Interception of mentorship

The third category highlighted the participants’ awareness of the pre-licensing year as one targeted at mentorship. Through reflecting on their experiences in allocated sites during community service, they identified under-resourced as an interceptor and actively engaged in thinking about alternate measures for future community service nurses.

#### Subcategory 3.1: Under-resourced

Participants cited a need for mentoring and working under supervision during their community service; however, that was not the case. They highlighted that an under-resourced working environment intercepted this community service goal of guidance. Participants felt pressurised during community service when expected to work independently as a member of the workforce instead of under supervision. The following quotes reveal the participants’ desire for support during community service despite the shortage of staff:

‘… We were reassured that during community service we will be supervised, mentored and supported, however this was not the case the clinical practice is so short-staffed we were always reminded of this and to work like anybody else ….’ (FG3, P1, Female)

‘… Midwifery units in most clinical facilities are short staffed, so as a community service midwife you are treated like a permanent…we were put under so much pressure … I felt we were robbed of that last chance of being supported as we developed into real clinical midwives.’ (FG4, P1, Female)

‘… I understand the shortage of staff but being put in the same pool with permanent staff was so unfair for us … our goal as community service midwives was to put our learnt theory into practice (with) the supervision of a midwife, not to be constantly reminded of shortage of staff.’ (FG2, P5, Female)

In addition, the participants were not rotated to other facets of maternity as they developed familiarity with a specific maternity setting, which some participants expressed as unjust exposure and prevented them from developing a more holistic approach to midwifery. These were some of the comments from the participants:

‘… The shortage of staff did not only rob us of the opportunity to be supported … but we were also not rotated to other units to learn more, we were told we will remain where we were.’ (FG1, P3, Male)

‘… We were told that the during community service we will be rotated to other units, but this was not the case, shortage of staff in the respective units we were in robbed us of this opportunity to rotate.’ (FG4, P4, Male)

‘I ended up working in labour ward for a long period of time, I did not get an opportunity to rotate to the postnatal unit because of shortage of staff.’ (FG5, P3, Female)

#### Subcategory 3.2: Active contemplation of community service goals

The participants acknowledged that their goals were unmet during community service; however, they contemplated employing strategies to improve future community service experiences. Participants highlighted the need for assistance and academic collaboration between the university and the clinical facilities, to improve newly qualified midwives’ experience in community service. This is supported by the following extracts:

‘My community service goals were not met but I feel something can be done to assist the students who are still to come after us, … in my thinking is that the university should work closely with the hospitals and ensure that our community service is pleasant.’ (FG1, P3, Male)

‘… If maybe the university can somehow collaborate with the … clinical areas that we going at, let them know that we are community … service … yes, we have graduated but we not yet ready to be part of the workforce, so they need to also supervise us they should not leave us …, alone and not supervised because we there to learn ….’ (FG4, P4, Male)

The following extracts show the further suggestion from the participants to strengthen their experience and learning by introducing an onsite named preceptor.

‘…It will be nice to have preceptors in the clinical facilities … someone specifically allocated to me as a newly qualified midwife in community service.’ (FGD1, P2, Male)

‘A named preceptor is what I would suggest ….’ (FGD4, P1, Female)

Participants also suggested amendments to the midwifery course, as indicated here.

‘… Midwifery course duration seemed not enough. It is only six months in the six months one must go to class, write examination, submit assignments and go to the clinical area, the time was so little.’ (FG4, P1, Female)

‘…Maybe doing it in a year at the same time with psychiatric nursing will be better.’ (FG2, P3, Female)

‘… At least to have some of the clinical examinations done in the clinical settings with real-life patients will be very helpful … the students will get exposure to what they will be most likely expected to do in the clinical world.’ (FG3, P2, Female)

‘… Yes, we can use the clinical skills laboratory, but the real-life exposure in terms of competencies is equally important to ensure that we are prepared for the real-life practice.’ (FG4, P4, Male)

## Discussion

In this study, the newly qualified midwives transitioning into the community service settings encountered situations which challenged their sense of being competent to practise in midwifery. The supportive environment of community service should ideally foster the beginnings of lifelong learning, engagement of higher-order thinking skills and enable newly qualified midwives to maximise their level of clinical competence as they adapt to their new roles requiring independent practice. Independence is important as skilled midwives can provide 87% of essential care needed for women and new-borns (Renfrew et al. 2014). The Novice to Expert theoretical model by Benner (1984) described five levels of progression in nurses’ and midwives’ competence, starting from a novice moving to an expert in the profession. Benner (1984) outlined the variances in the different levels for receiving support and guidance versus the provision thereof. As regulated by the SANC (RSA 2006), mandatory community service is a critical period to assist the novice to move to an advanced beginner within a safe learning environment. However, contrary to the SANC’s goals the initial introduction to community service was found to be stressful for the newly qualified midwives, transitioning from what they perceived to be the sheltered education environment.

The newly qualified midwives struggled to adapt to their new roles, which required that they merged their professional ideals with the reality of the practice world of midwifery that demanded accountability. In their struggles, they experienced transitional insecurities, which align with ‘transition shock’, as described by Duchscher (2009). In an attempt to prepare its graduates, the Nursing Discipline of the study setting promotes the development of cross-discipline skills couched in activities aimed at developing higher-order thinking skills (Amod & Brysiewicz 2019; Baloyi & Mtshali 2018; Jarvis & Baloyi 2020), with predominantly supervised authentic real-life experiences. However, in their anxiety, the participants appeared unable to embrace their learning and failed to value the nursing discipline’s context-driven curriculum. The participants had trouble transferring the content learned in the simulated setting during their university education, into the real practice world, thus describing their clinical education and assessments as unrelatable to the midwifery clinical practice. Schytt and Waldenström (2013) shared similar findings. They highlighted that newly qualified midwives encountered difficulty in confidently executing midwifery services because of the incompatibility between the unfamiliar clinical setting and their familiar simulated university environment. Similarly, Benner (1984) described the need to recognise that the novice has no experience to make decisions when confronted with real-life incidents and tends towards strict adherence of guidelines and formally taught practice when problem-solving, thus restricting the range of their nursing interventions.

The novice has limited clinical experience and requires guidance and support from those described by Benner (1984) as competent, proficient and experts. As a result of the high level of anxiety, the participants discussed support as the most valuable factor, whilst the disparate realities were seen as polar opposite experiences of support and the stigmatising approaches emerged as core hindrances. The lack of support further exacerbated the lack of confidence and doubts expressed by the newly qualified midwives. Support was not a consistent guarantee but dependant on who was on duty, described in gambling terms as ‘luck of the draw’ as to who received a nurturing, hand-holding mentor or who was ‘expected to adapt or die’. Benner (1984) discussed that support from senior colleagues is necessary for nurses and midwives to move to the next level of competence. Vygotsky (1978) identified the significance of support to allow for movement from the zone of proximal development to more independent functioning. Studies have shown that newly qualified midwives value the eager sharing of knowledge, expertise (Fenwick et al. 2012) and support, which are crucial in their professional development (Simane-Netshisaulu & Maputle 2019). However, the registered midwives’ stigmatisation towards the newly qualified midwives as being ‘all-knowing’ left them feeling intimidated and reluctant to seek guidance from the senior staff for fear of being labelled as incompetent and treated unfairly. The ability to actively liaise around care and ask questions without feeling judged is a major strategy that contributes to a sense of being supported and feeling safe (Pairman et al. 2016).

Bond and Holland (2011) argued that constant and direct supervision is the most fruitful approach to improve clinical competence. However, the newly qualified midwives experienced an interruption in their supervision and support, hindering the achievement of the community service goals. The midwifery units were understaffed and therefore, the institution’s needs determined the newly qualified midwives’ rotation through the midwifery units instead of consideration for their needs for growth and development, which could have helped them move towards Benner’s (1984) next level of competence. Rotations through all the different maternity areas offer key learning opportunities during the transition period (Clements 2012).

Despite the majority of the participants experiencing transitional insecurities and acknowledging that their community service goals were unmet, they contemplated measures to improve future community service experiences and skills development for newly qualified midwives. They made two suggestions, firstly strengthening the collaboration between the university and clinical facilities, extending conversations to include the needs of a newly qualified midwife, and secondly, onsite facilitation by a named preceptor. A named preceptor can contribute to allaying anxiety, increasing confidence and competence and job satisfaction (Steele 2009). The participants’ suggestion concurred with Simane-Netshisaulu and Maputle (2019) who identified the significance for the beginning practitioner to work alongside experienced staff and observe, learn and practice to make the most of available learning opportunities. These suggestions could counter the Saving Mothers Report (SANDoH 2018) findings, which stated that the lack of appropriately trained midwives is the most frequently cited avoidable factor related to a high MMR.

### Limitations

The study held two limitations, namely that the setting was limited to one geographical area (eThekwini District) and not all of the province, as a result, findings are generalisable upon consideration; secondly participants were from one university.

### Recommendations

The specific assignment of clinical facilitators to support and supervise newly qualified midwives. The midwives are rotated through all maternity units during their community service.

The meeting between nursing academia and midwives needs to be strengthened by including discussion on the transition of the newly qualified midwives from the academic setting to the practice setting. The agenda of such meetings should be to organise workshops for qualified midwives to orientate them to the goals of the community service midwives, re-looking at their supportive role and reflecting on stigmatising attitudes.

Despite students’ authentic real-life experiences, in the students’ education there are repeated reminders of the relevance of all undergraduate learnings for the real-life practice world post-qualifying.

A suggested future study to identify diploma qualified nurses and midwives’ perceptions of their colleagues educated through university programmes.

## Conclusion

Newly qualified midwives’ clinical confidence and competence transitioning from the safe academic environment to the authentic accountable clinical setting hinges on welcoming non-stigmatising supportive relationships. The relationships facilitate the integration of previous learnings into community service practice, which hold significance as midwives form the backbone of maternal and neonatal health. Inadequately prepared midwives highlight with concern the possible interruption of global (UN 2015) and national health priorities (RSA 2011). Hence, critical to developing competent midwives is to strengthen the new graduate’s transitioning phase into the profession. This study can create a better understanding of the experience of new graduate midwives and how to effectively educate them during their pre-service courses, fostering smooth transitions to the workplace setting.
